# Patient preferences in geriatric wards, a survey of health care professionals’ practice, experience and attitudes

**DOI:** 10.1007/s41999-023-00922-7

**Published:** 2024-01-29

**Authors:** Hege Ihle-Hansen, R. Pedersen, S. F. Westbye, T. J. L. Sævareid, L. Brøderud, M. H. Larsen, K. Hermansen, S. Rostoft, M. Romøren

**Affiliations:** 1 Centre for Medical Ethics, Institute of Health and Society, Faculty of Medicine, University of Oslo, Oslo, Norway; 2 Institute for Clinical Medicine, Faculty of Medicine, University of Oslo, Oslo, Norway; 3 Department of Neurology, Oslo University Hospital, Oslo, Norway; 4 Lovisenberg Diaconal University College, Oslo, Norway; 5 Department of Health Sciences, Faculty of Medicine and Health Sciences, Norwegian University of Science and Technology, Aalesund, Norway; 6 Department of Geriatric Medicine, Oslo University Hospital, Oslo, Norway

**Keywords:** Patient preferences, Person-centered, Shared decision-making, Advance care planning

## Abstract

**Purpose:**

We aimed to identify whether health care professionals (HCP) examine their patient and next-of-kin preferences, and to study whether medical decisions follow these preferences.

**Method:**

A cross-sectional web-based survey was conducted with multidisciplinary HCP from 12 geriatric wards in the South-Eastern Norway Regional Health Authority.

**Results:**

Of the 289 HCPs responding (response rate 61%), mean age 37.8 years (SD 11.3), 235 (81.3%) women, 12.4 (SD 9.6) years of experience and 67 (23.2%) medical doctors, only half report clarifying patients’ preferences. The majority reported that they did not inform, involve and treat in line with such preferences. However, 53% believe that HCP, patients and next-of-kin should make clinical decisions together.

**Discussion:**

Our findings indicate a lack of engagement in conversation and inclusion of patient preferences when providing health interventions in geriatric wards. Measures for change of culture are needed.

**Supplementary Information:**

The online version contains supplementary material available at 10.1007/s41999-023-00922-7.

## Key summary points

**Aim:**

Medical doctors making decisions without consulting their patients has gradually shifted towards shared decision making.

**Findings:**

Nevertheless, in this study from geriatric wards in Norway, only half of health care professionals (HCP) report that patient preferences were clarified, and the majority of HCP reported that they did not inform, involve, and treat patients based on patient preferences.

**Message:**

Measures are needed to improve integration of patient’s preferences into decision-making.

**Supplementary Information:**

The online version contains supplementary material available at 10.1007/s41999-023-00922-7.

## Introduction

High quality, person-centered medical care incorporates patient preferences, values, and goals for shared ownership in the decision-making process [1–4].

Patient preferences for information, involvement, diagnostics, treatment, self-management, care and end-of-life capture ethical considerations and a legal framework respecting patient autonomy [3].

Health care professionals (HCP) working in geriatric wards are trained to assess patient preferences and provide coordinated healthcare through a multidisciplinary team approach [2] and could, therefore, be considered more prone to address and follow patients’ preferences. Historically, the political and legal shift from paternalism to patient’s autonomy was introduced through the informed consent in health law, also in Norway. However, most laws in Europe including Norway still give the medical doctor (MD) the final say, challenging patient’s autonomy in medical decisions. Only a handful of countries outside the US recognize patient’ wishes as legally binding by the use of advance directives. During the past decade, attempts have been conducted to empower patients’ and families’ autonomy in medical decisions by the use of evidence-based communication tools to increase patients involvement, such as advance care planning (ACP) and shared decision-making (SDM) [5–8].

Still, the recent literature indicates ACP has severe implementation problems with barriers such as inconsistency in effect on goal-concordant care and less integration in wards for patients admitted with acute severe illness [5, 9]. In this study, prior to a randomized trial implementing ACP in geriatric wards, we aimed to identify to what extend HCP examine the patient and next-of kin preferences for information, involvement, and treatment and to synthesize the experience of clinical practice in accordance with these preferences. Finally, we assessed possible causes for discrepancies and HCP’s attitudes towards clinical decision-making.

## Methods

We invited HCP working ≥20% in 12 geriatric wards in hospitals in the Southeastern region of Norway to a survey to explore HCP`s self-reported practice and attitudes for assessing patient preferences, prior to implementation of ACP. The participating hospitals had acute geriatric wards or internal medical wards with a small number of beds allocated to geriatric patients and with a geriatrician among the staff.

We conducted an anonymous, descriptive, cross-sectional web-based survey, inspired by others [10, 11] (Table 1 Supplementary). The recruitment period was four weeks from 18th of October 2022. The questionnaire consisted of four sections related to three different categories of patient preferences; information, involvement, and treatment. First, we examined whether HCP clarify the patient preferences. Second, we investigated the experiences of clinical practice in accordance with these preferences. Third, we asked for possible reasons for not informing or involving patients or next- of-kin. We also asked for HCP confidence in knowing the patient preferences for involvement (VAS scale 0–10). In the fourth section, we explored HCP’s attitudes towards who makes and who should make clinical decisions. We recorded HCP’s age, sex, profession, years in clinical practice and experience as a leader.

**Table 1:** Demographic and background characteristics of the 289 participants from 12 geriatric units in South-Eastern Norway Regional Health Authority

	*n*	%
Age		
20–29	92	(32)
30–39	81	(28)
40–49	66	(23)
50–59	37	(13)
60–66	13	(4)
Sex		
Female	235	(81)
Years in practice		
0–5 years	85	(29)
10-Jun	66	(23)
15-Nov	59	(17)
16–20	31	(11)
>20	56	(19)
Health profession		
Medical doctor, not specialized	20	(7)
Internist	6	(2)
Geriatrician	41	(14)
Nurse	131	(45)
Special trained nurse	25	(9)
Assistant nurse	35	(12)
Physiotherapist/occupational therapist	31	(11)
Experience as a leader	24	(8)

Each survey participant gave written consent. Norwegian Centre for Research Data, the Data Protection Official for Research, approved the study (805491). The study is covered by the Act on medical and health research according to the Regional Ethical Committee and has been approved by local data protection officers at each trial site. The responses were stored at the University of Oslo’s Services for Sensitive Data.

### Statistics

We report continuous variables as mean and standard deviation (SD) and categorical variables as frequencies and percentage. We dichotomized “clarify for patient preferences for treatment” into always/often versus sometimes/never and “experienced practice” into following (response “in line with”) versus not following the preferences (response not known, less than wanted and more than wanted) and compared the groups using Chi-square test. We used SPSS version 29.

## Results

All 12 eligible hospitals agreed to participate. After inviting 470 HCP, 289 responded (response rate 61%), with mean age 37.8 years (SD 11.3), 235 (81.3%) women, 12.4 (SD 9.6) years of experience and 67 (23.2%) MDs (Table 1).

### Clarification of preferences

When examining HCP, 192 (66.4%) sometimes/never clarified the patient preferences for information, 160 (55.4%) for involvement and 125 (43.3%) for treatment. HCP reported that 178 (61.6%) and 142 (49.1%) sometimes/never clarify preferences for information and involvement of next-of-kin (Table 2 and Table 2 Supplementary). The results did not change when we stratified for years of practice.

**Table 2:** Health care professionals’ assessments of patient preferences and attitudes to decision making, n (%)

	*N* (%)		*N* (%)
*Health care professionals’ assessment of patient’s preferences*			
Clarify preferences for information		Experience of clinical practice in accordance with	
Patients’ preference for information		Patients’ preference for information	
Always/often	97 (33.6)	In line with	98 (33.9)
Sometimes/never	192 (66.4)	Not following the preferences	191 (66.1)
Patients’ preference for information to next of kin		Patients’ preference for information to next of kin	
Always/often	139 (48.1)	In line with	193 (66.8)
Sometimes/never	158 (21.9)	Not following the preferences	96 (66.8)
Next of kin preference for information		Next of kin preference for information	
Always/often	111 (38.4)	In line with	170 (58.8)
Sometimes/never	178 (61.6)	Not following the preferences	119 (41.2)
Clarify preferences for involvement		Experience of clinical practice in accordance with	
Patients’ preference for involvement		Patients’ preference for involvement	
Always/often	129 (44.6)	In line with	135 (46.7)
Sometimes/never	160 (55.4)	Not following the preferences	154 (53.3)
Patients’ preference for involvement of next of kin		Patients’ preference for information to next of kin	
Always/often	140 (48.4)	In line with	182 (63.0)
Sometimes/never	149 (51.6)	Not following the preferences	107 (37.0)
Next of kin preference for involvement		Next of kin preference for information	
Always/often	147 (50.9)	In line with	159 (55.0)
Sometimes/never	142 (49.1)	Not following the preferences	130 (45.0)
Clarify patients’ preference for treatment	164 (56.7)	Experience of clinical practice in accordance with patients’ preference for treatment	138 (47.8)
	125 (43.3)		151 (52.2)
*Health care professionals’ attitudes to decision-making*			
Attitudes to who makes the decisions		Attitudes to who should make clinical decisions	
HCP	61 (21.1)	HCP	13 (4.5)
Patients	8 (2.8)	Patients	15 (5.2)
Next-of-kin	5 (1.7)	Next-of-kin	0
Patients and next –of-kin	7 (2.4)	Patients and next –of-kin	10 (3.5)
HCP and patients	101 (34.9)	HCP and patients	89 (30.8)
HCP and next-of-kin	20 (6.9)	HCP and next-of-kin	7 (2.4)
HCP, patients and next-of-kin	74 (25.6)	HCP, patients and next-of-kin	153 (52.9)
Do not know	13 (4.5)	Do not know	2 (0.7)

*HCP* health care professionals

### Inclusion of patient preferences in clinical practice

Of 289, 191 (66.1%) of HCP do not follow patient preferences for information either because patient preferences are unknown, or they choose to inform more or less than wanted. Further, 154 (53.9%) do not follow the preferences for involvement and 151 (52.2%) do not follow the preferences for treatment, and similar trends are reported towards next-of-kin (Table 2).

### Discrepancy with norms and clinical practice

Reasons stated for not *informing* or *involving* patients were (1) not enough resources or time [180 (62.3%) for informing and 167 (57.8%) for involving], (2) patients being too sick [99 (34.3%) and 116 (40.1%)], and (3) the need for better knowledge and communication skills [97 (33.6%) and 105 (36.3%)], respectively. Other reasons were short hospital stays [72 (24.8%) and 66 (22.8%)] and limited access to single rooms [67 (23.2%) and 50 (17.3%)]. The median score for confidence in knowing the patient preferences for involvement was 6.0 (5–8) (Fig. 1 Supplementary display the distribution).

Only 115/164 HCP (70.1%) follow the patient preferences for treatment. Interestingly, 23/125 (18.4%) report conducting treatment in accordance with the patient preferences without clarifying them (Table 3), with significant differences in responses regarding clarifying preferences and experience of clinical practice (*P* < 0.01). In subgroup analyses with MD we found the same trends.

**Table 3:** The relationship between clarifying the patient preferences for treatment and experienced practice of following the preferences

			Clarify		
			Always/often	Sometimes/never	*P*
					*P* < 0.001
Experienced practice	All HCP	Following	115 (70.1)	23 (18.4)	
		Not following	49 (29.9)	102 (81.6)	

*HCP*  health care professionals

### Attitudes to decision-making

In clinical practice, 61 (21.1%) report that HCP make the decisions alone, while only 13 (4.5%) think they should. Interestingly, 153 (52.9%) think that HCP, patients and next-of-kin should make decisions together, while only 74 (25.6%) report this to be integrated into their clinical practice (Table 2).

## Discussion

We found that even in geriatric wards more prone to a person-centered approach, only half of HCP report clarifying patient preferences for information, involvement, and treatment. Further, more than half do not inform, involve, and treat in line with these preferences, and only 70% report they follow the clarified preferences for treatment. Lack of resources, time, knowledge, communication skills and confidence and too sick patients are some reported reasons for not discussing preferences with the patient. Few HCP think that they alone should make clinical decisions, while more than 50% report that HCP, patients and next-of-kin should make decisions together.

Considering the attempts to strengthen patient’s autonomy, the results indicate a lack of impact and that action is needed to empower patients and families in the health care system. This is of relevance for Norway with an even more paternalistic legal framework than neighboring countries, also for most countries since very few have legally binding obligations to patients.

When failing to clarify patient preferences, we may provide healthcare that is not goal-concordant and not complying with their rights for participation in decision-making processes [12]. In older adults, frailty and comorbidity frequently lead to a higher risk of complications, and individual patient priorities play a particularly important role in decision-making. Through conversations with patients about their preferences, we might ensure that patients understand their disease and prognosis, acknowledge their right to participate, and reduce both over-and under diagnosis and treatment. Involving next-of-kin allows us to ensure that patients` wishes are known [13].

HCP experience that they inform, involve, and treat in accordance with patient preferences, even without clarifying such preferences. This may reflect a persistent paternalistic and provider-orientated culture in the wards, or more focus on diagnosis and treatment than the (actual) problems experienced by the patient [4, 10, 14]. When one-third of the HCP report clarifying the patient preferences and still do not adapt the treatment accordingly, we can assume an experience of moral stress and a gap in patient and HCP’s expectations of treatment opportunities. If this is the case, there is an urgent need to develop a culture for educating and encouraging communication for assessing preferences, both for immediate and future care [15]. Proposed strategies for improvement at a clinical level are to normalize ACP conversations, team-discussion of prognosis and training in communication skills for quality and trust in clinician-patients conversations, especially in acute severe illness where the decisions have high impact [5, 6, 15, 16]. Further, there is a need for leadership and operational plans at organizational- and system-levels [9, 16].

Resources and time, severely ill patients and the need for more knowledge, communication skills and confidence are known also previously reported causes for not informing and involving according to wishes of the patient and next-of-kin [17]. Other possible explanations can be older patients with impaired cognitive function and complex and severe conditions with prognostic uncertainty [2]. Health literacy (the degree of having the capacity to obtain, process, and understand basic health information and services needed to make appropriate health decisions,) is also known to be low among older patients [18].

HCP in our survey seems to favor shared decision-making between HCP, patients, and next-of-kin. Assessing preferences is valuable [7, 8], even in the context of acute hospitalization. Further, patients in geriatric wards suffer high morbidity and mortality, with frequent decisions about limit life-sustaining treatment, where next-of-kin often can inform about the patient preferences and priorities in life. Even if Norway and most European countries do not have advance directives, patient views should always be considered. This underscores the importance of having access to the patient’s views, where ACP could be an important source [19, 20].

One strength of the current study is the high number of respondents from different hospitals to elicit perspectives of important ethical and legal principles. The survey methodology has limitations with self-reported design, non-validated measures, lack of information of non-responders and other explanations for discrepancies, subjective experiences with personal opinions, not assessing goals and values or access to medical records.

In conclusion, our findings support a limited engagement in conversation and inclusion of patient preferences when providing healthcare to older patients in acute geriatric wards. There is an urgent need to develop a culture for respecting autonomy by involving patients and their next-of-kin into ethical healthcare discussions with a more personalized thinking. Clinicians may consider using ACP and develop communication skills to integrate patient’s autonomy when providing medical care.

### Supplementary Information

Below is the link to the electronic supplementary material.

Supplementary file1 (DOCX 69 kb)

Supplementary file2 (DOCX 16 kb)

Supplementary file3 (DOCX 19 kb)

## References

[1] Charles C, Gafni A, Whelan T (1997). Shared decision-making in the medical encounter: what does it mean? (or it takes at least two to tango). Soc Sci Med.

[2] Stuck AE, Masud T (2022) Health care for older adults in Europe: how has it evolved and what are the challenges? Age Ageing 51(12):afac287 10.1093/ageing/afac287 PMC9760902 36529998

[3] Gonzalez AG, Schmucker C, Nothacker J, Motschall E, Nguyen TS, Brueckle MS (2019). Health-related preferences of older patients with multimorbidity: an evidence map. BMJ Open.

[4] Rostoft S, van den Bos F, Pedersen R, Hamaker ME (2021). Shared decision-making in older patients with cancer—what does the patient want?. J Geriatr Oncol.

[5] Jimenez G, Tan WS, Virk AK, Low CK, Car J, Ho AHY (2018). Overview of systematic reviews of advance care planning: summary of evidence and global lessons. J Pain Symptom Manage.

[6] van der Horst DEM, Garvelink MM, Bos WJW, Stiggelbout AM, Pieterse AH (2023). For which decisions is shared decision making considered appropriate?—a systematic review. Patient Educ Couns.

[7] Sudore RL, Lum HD, You JJ, Hanson LC, Meier DE, Pantilat SZ (2017). Defining advance care planning for adults: a consensus definition from a multidisciplinary Delphi panel. J Pain Symptom Manage.

[8] Rietjens JAC, Sudore RL, Connolly M, van Delden JJ, Drickamer MA, Droger M (2017). Definition and recommendations for advance care planning: an international consensus supported by the European Association for Palliative Care. Lancet Oncol.

[9] Westbye SF, Rostoft S, Romøren M, Thoresen L, Wahl AK, Pedersen R (2023). Barriers and facilitators to implementing advance care planning in naïve contexts—where to look when plowing new terrain?. BMC Geriatr.

[10] Murtagh FE, Thorns A (2006). Evaluation and ethical review of a tool to explore patient preferences for information and involvement in decision making. J Med Ethics.

[11] Van Scoy LJ, Day AG, Howard M, Sudore R, Heyland DK (2019). Adaptation and preliminary validation of the advance care planning engagement survey for surrogate decision makers. J Pain Symptom Manage.

[12] Etkind SN, Bone AE, Lovell N, Higginson IJ, Murtagh FEM (2018). Influences on care preferences of older people with advanced illness: a systematic review and thematic synthesis. J Am Geriatr Soc.

[13] Malhotra C, Huynh VA, Shafiq M, Batcagan-Abueg APM (2022) Advance care planning and caregiver outcomes: intervention efficacy—systematic review. BMJ Support Palliat Care. 10.1136/spcare-2021-003488 10.1136/spcare-2021-003488 35788465

[14] Combes S, Nicholson CJ, Gillett K, Norton C (2019). Implementing advance care planning with community-dwelling frail elders requires a system-wide approach: an integrative review applying a behaviour change model. Palliat Med.

[15] Rosa WE, Izumi S, Sullivan DR, Lakin J, Rosenberg AR, Creutzfeldt CJ (2023). Advance care planning in serious illness: a narrative review. J Pain Symptom Manage.

[16] Nortje N, Zachariah F, Reddy A (2023). Advance Care Planning conversations: what constitutes best practice and the way forward: Advance Care Planning-Gespräche: Was Best Practice ausmacht und wie es weitergehen kann. Z Evid Fortbild Qual Gesundhwes.

[17] Fien S, Plunkett E, Fien C, Greenaway S, Heyland DK, Clark J (2021). Challenges and facilitators in delivering optimal care at the End of Life for older patients: a scoping review on the clinicians’ perspective. Aging Clin Exp Res.

[18] Chesser AK, Keene Woods N, Smothers K, Rogers N (2016). Health literacy and older adults: a systematic review. Gerontol Geriatr Med.

[19] Wikstøl D, Pedersen R, Magelssen M (2021) Public attitudes and health law in conflict: somatic vs. mental care, role of next of kin, and the right to refuse treatment and information. BMC Health Serv Res 21(1):3 10.1186/s12913-020-05990-0 PMC7780687 33390168

[20] Sævareid TJL, Pedersen R, Magelssen M (2021). Positive attitudes to advance care planning—a Norwegian general population survey. BMC Health Serv Res.

