# Supplementary material for: Patient preferences in geriatric wards, a survey of health care professionals’ practice, experience and attitudes
Source: Eur Geriatr Med. 2024 Jan 29;15(1):153–8. doi: 10.1007/s41999-023-00922-7 (PMC10876711; doi:10.1007/s41999-023-00922-7)
Supplement: Supplementary file 1 — Supplementary file1 (DOCX 69 kb) [file 41999_2023_922_MOESM1_ESM.docx]

Figure 1. Confidence in knowing patient preference for involvement in decision-making, n=289


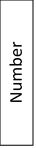

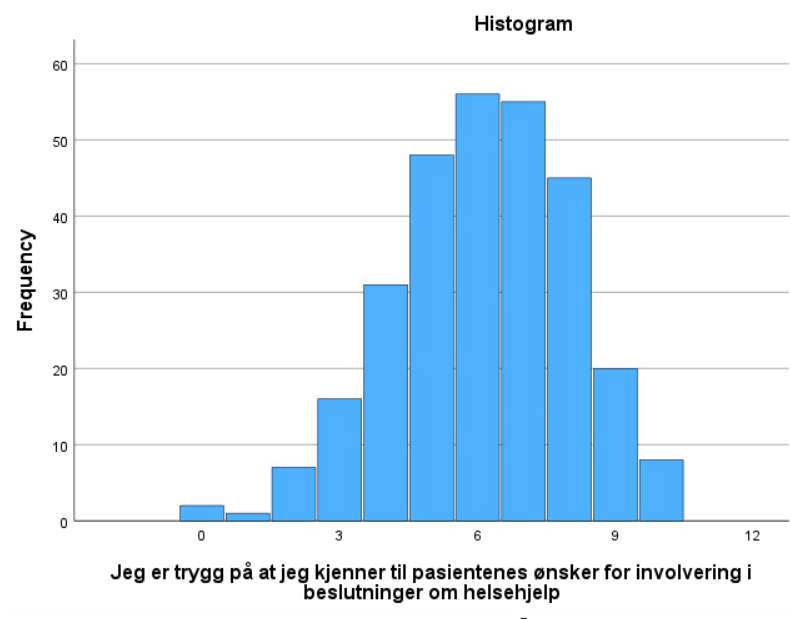


| VAS scale | 0 | 1 | 2 | 3 | 4 | 5 | 6 | 7 | 8 | 9 | 10 |
| --- | --- | --- | --- | --- | --- | --- | --- | --- | --- | --- | --- |
