# Supplementary material for: Patient preferences in geriatric wards, a survey of health care professionals’ practice, experience and attitudes
Source: Eur Geriatr Med. 2024 Jan 29;15(1):153–8. doi: 10.1007/s41999-023-00922-7 (PMC10876711; doi:10.1007/s41999-023-00922-7)
Supplement: Supplementary file 2 — Supplementary file2 (DOCX 16 kb) [file 41999_2023_922_MOESM2_ESM.docx]

Table 1, Supplementary. Survey questions to assess how health care professionals consider the patient preferences

|  | Clarification of preferences | Experience of current practice | Reasons for practice | Attitudes to assessments |
| --- | --- | --- | --- | --- |
| Information | Clarify the patients preferences for information  1-Always  2-Often  3-Sometimes  4-Never | To inform the patient in accordance with their preferences  1-Patient not informed  2-Not assessed  3-In line  4-Less than wanted  5-More than wanted | 1- Not enough resources or time  2-Patient are to seek  3-More knowledge and communication skills are needed  4-Too short length of stay  5-No access to single rooms | Clinical decision making is done by  1-HP  2-Patients  3-Next-of-kin  4-Patient and next-of-kin  5-HP and patients  6-HP and next-of-kin  7-HP and next-of-kin  HP, patient and next-of-kin  8-Do not know  Who should make clinical decisions  1-HP  2-Patients  3-Next-of-kin  4-Patiens and next-of-kin  5-HP and patient  6-HP and next-of-kin  7-HP, patient and next-of-kin  8-Do not know |
|  | Clarify the patients preferences for information to next of kin  1-Always  2-Often  3-Sometime  4-Never | To inform the next of kin in accordance with the patient preferences  1-Next of kin not informed  2-Not assessed  3-In line  4-Less than wanted  5-More than wanted |  |  |
|  | Clarify the next of kin preference for information  1-Always  2-Often  3-Sometime  4-Never | To inform the next of kin in accordance with their preferences  1-Next of kin not informed  2-Not assessed  3-In line  4-Less than wanted  5-More than wanted |  |  |
| Involvement | Clarify patients preferences for involvement in decision making  1-Always  2-Often  3-Sometime  4-Never | To involve the patient in medical decision making in accordance with the preferences  1-Patient not involved  1-Not assessed  2-In line  3-Less than wanted  4-More than wanted | 1-Not enough resources or time  2- Patient are too seek  3- More knowledge and communication skills are needed  4-Too short length of stay  5-No access to single room  Do you feel confident you know your patient’s preferences for involvement in medical decisions  Scale 0-10 |  |
|  | Clarify patients preferences for involvement of next of kin  1-Always  2-Often  3-Sometime  4-Never | To involve the next of kin in medical decision making in accordance with the patient preferences  1-Next of kin not informed  2-Not assessed  3-In line  4-Less than wanted  5-More than wanted |  |  |
|  | Clarify the next of kin preference for involvement  1-Always  2-Often  3-Sometime  4-Never | To involve the next of kin in medical decision making in accordance with their preferences  1-Next of kin not informed  2-Not assessed  3-In line  4-Less than wanted  5-More than wanted |  |  |
| Treatment | Clarify patients preferences for given treatment  1-Always  2-Often  3-Sometimes  4-Never | To treat the patient in accordance with the preferences  1-Not assessed  2-In line  3-Less than wanted  4-More than wanted |  |  |
