# Supplementary material for: Patient preferences in geriatric wards, a survey of health care professionals’ practice, experience and attitudes
Source: Eur Geriatr Med. 2024 Jan 29;15(1):153–8. doi: 10.1007/s41999-023-00922-7 (PMC10876711; doi:10.1007/s41999-023-00922-7)
Supplement: Supplementary file 3 — Supplementary file3 (DOCX 19 kb) [file 41999_2023_922_MOESM3_ESM.docx]

| **Health care professionals’ assessment of patient’s preferences** | | | | | | | | | | | | | |
| --- | --- | --- | --- | --- | --- | --- | --- | --- | --- | --- | --- | --- | --- |
| N=289 | **Preferences** | **Information** | | | | **Involvement** | | | | | **Treatment** | | |
| **HCP** | **Response** | **Patient** | | **Patient preferences for next of kin** | **Next of kin** | **Patient** | | | **Patient for next of kin** | **Next of kin** | **Patient** | | |
| **Clarify** | Always | 7 (2.4) | 97 (33.6) | 28  (9.7) | 14 (4.8) | 16  (5.5) | 129 (44.6) | | 22  (7.6) | 21 (7.3) | 25  (8.7) | 164 (56.7) | |
|  | Often | 90 (31.1) |  | 111 (38.4) | 97 (33.6) | 113 (39.1) |  |  | 118 (40.8) | 126 (43.6) | 139 (48.1) |  |  |
|  | Sometimes | 171 (59.2) | 192 (66.4) | 139 (48.1) | 158 (54.7) | 150 (51.9) | 160 (55.4) | | 145 (50.2) | 136 (47.1) | 120 (41.5) | 125 (43.3) | |
|  | Never | 21  (7.3 ) |  | 11 (3.8) | 20 (6.9) | 10  (3.5) |  |  | 4  (1.4) | 6 (2.1) | 5  (1.7) |  |  |
| **Experience** | In line with | 98 (33.9 ) | 98 (33.9) | 193 (66.8) | 170 (58.8) | 135 (46.7) | 135 (46.7) | | 182 (63.0) | 159 (55.0) | 138 (47.8) | 138 (47.8) | |
|  | Not known | 3  (1.0 ) | 191 (66.1) | 1  (0.3) | 0 | 21  (7.3) | 154 (53.3) | | 9  (3.1) | 3 (1.0) | No | 151 (52.2) | |
|  | Not assessed | 142 (49.1) |  | 87 (30.1) | 82 (28.4) | 82 (28.4) |  |  | 80 (27.7) | 86 (29.8) | 78  (27.0) |  |  |
|  | Less than wanted | 40 (13.8) |  | 6  (2.1) | 34 (11.8) | 50 (17.3) |  |  | 12  (4.2) | 37 (12.8) | 15  (5.2) |  |  |
|  | More than wanted | 6  (2.1) |  | 2  (0.7) | 3 (1.0) | 1  (0.3) |  |  | 6  (2.1) | 4 (1.4) | 58  (20.1) |  |  |
|  | **Health care professionals’ attitudes to decision making** | | | | | | | | | | | | |
| **Attitudes** | **Response** | | | **Who makes the decisions** | | | | | **Who should make the decisions** | | | | |
|  |  | | | **All HCP** | | | | **MD** | **All HCP** | | | | **MD** |
|  | HCP | | | 61 (21.1 ) | | | | 26 (38.8) | 13 (4.5) | | | | 7 (10.4) |
|  | Patients | | | 8 (2.8) | | | | 1  (1.5) | 15 (5.2) | | | | 2(3.0) |
|  | Next-of-kin | | | 5 (1.7) | | | | 0 | 0 | | | | 0 |
|  | Patients and next –of-kin | | | 7 (2.4) | | | | 0 | 10 (3.5) | | | | 0 |
|  | HCP and patients | | | 101 (34.9) | | | | 27 (42.2) | 89 (30.8) | | | | 25 (37.3) |
|  | HCP and next-of-kin | | | 20 (6.9) | | | | 3  (4,5) | 7 (2.4) | | | | 0 |
|  | HCP, patients and next-of-kin | | | 74 (25.6) | | | | 10  (14.9) | 153 (52.9) | | | | 32 (47.8) |
|  | Do not know | | | 13 (4.5) | | | | 0 | 2 (0.7) | | | | 1 (1.5) |

Table 2 Supplemetary. Health care professionals’ assessments of patient’s preferences and attitudes to decision m

HCP= Health care professionals; MD=Medical doctoraking, n (%)
